# Hospital-level variation in hospitalization costs for spinal fusion in the United States

**DOI:** 10.1371/journal.pone.0298135

**Published:** 2024-02-08

**Authors:** Joanna Curry, Nam Yong Cho, Shannon Nesbit, Shineui Kim, Konmal Ali, Varun Gudapati, Richard Everson, Peyman Benharash

**Affiliations:** 1 Cardiovascular Outcomes Research Laboratories (CORELAB), David Geffen School of Medicine, University of California, Los Angeles, CA, United States of America; 2 Department of Surgery, David Geffen School of Medicine, University of California, UCLA, Los Angeles, CA, United States of America; 3 Department of Neurosurgery, David Geffen School of Medicine, University of California, Los Angeles, CA, United States of America; Tokyo Medical and Dental University (TMDU), JAPAN

## Abstract

**Background:**

With a growing emphasis on value of care, understanding factors associated with rising healthcare costs is increasingly important. In this national study, we evaluated the degree of center-level variation in the cost of spinal fusion.

**Methods:**

All adults undergoing elective spinal fusion were identified in the 2016 to 2020 National Inpatient Sample. Multilevel mixed-effect models were used to rank hospitals based on risk-adjusted costs. The interclass coefficient (ICC) was utilized to tabulate the amount of variation attributable to hospital-level characteristics. The association of high cost-hospital (HCH) status with in-hospital mortality, perioperative complications, and overall resource utilization was analyzed. Predictors of increased costs were secondarily explored.

**Results:**

An estimated 1,541,740 patients underwent spinal fusion, and HCH performed an average of 9.5% of annual cases. HCH were more likely to be small (36.8 vs 30.5%, p<0.001), rural (10.1 vs 8.8%, p<0.001), and located in the Western geographic region (49.9 vs 16.7%, p<0.001). The ICC demonstrated 32% of variation in cost was attributable to the hospital, independent of patient-level characteristics. Patients who received a spinal fusion at a HCH faced similar odds of mortality (0.74 [0.48–1.15], p = 0.18) and perioperative complications (1.04 [0.93–1.16], p = 0.52), but increased odds of non-home discharge (1.30 [1.17–1.45], p<0.001) and prolonged length of stay (β 0.34 [0.26–0.42] days, p = 0.18). Patient factors such as gender, race, and income quartile significantly impacted costs.

**Conclusion:**

The present analysis identified 32% of the observed variation to be attributable to hospital-level characteristics. HCH status was not associated with increased mortality or perioperative complications.

## Introduction

Spinal fusion is a commonly utilized surgical procedure to treat a broad range of spinal conditions including degenerative spondylolisthesis, radiculopathy, and discogenic pain. The overall 118% increase in the volume of spinal fusions across the US between 1990 and 2014, has been attributed to surgical advances in the field as well as the aging population [1]. Notably, hospital charges for such procedures have nearly tripled over the same period paralleling an annual growth rate of nearly 20% for the spinal implant industry [2,3]. As such, the cost efficiency and utilization patterns of spinal fusion have come under scrutiny. With the rising incidence of patients undergoing spinal fusion, examination of utilization practices and the financial impact on the healthcare system is particularly relevant.

The introduction of value-based healthcare delivery in the United States has motivated a shift in reimbursement paradigms towards improving patient outcomes and reducing the overall costs of care [4,5]. Although spinal fusion is not currently included in value-based models, it may be a suitable candidate for such approaches [6]. Despite the annual charges for such procedures surpassing $48 billion in 2014, factors contributing to the stark increase in cost remain poorly understood [1]. In a single-center study, Twitchell et. al reported 36% of the total costs for spinal fusion to be attributable to facility costs [7]. However, these results are not generalizable and center-level variation could not be examined as only one hospital was included in the analysis. In the absence of standardized protocols including Enhanced Recovery After Surgery (ERAS) programs, the presence of significant inter-hospital variation in costs of spinal fusion is plausible.

The present study used a national database to examine center-level variation in risk-adjusted episodic costs of spinal fusion across the United States. We hypothesized the presence of significant disparities in inpatient costs with high-cost hospitals yielding similar patient outcomes to other centers.

## Materials and methods

### Data source and study population

**Fig 1:**
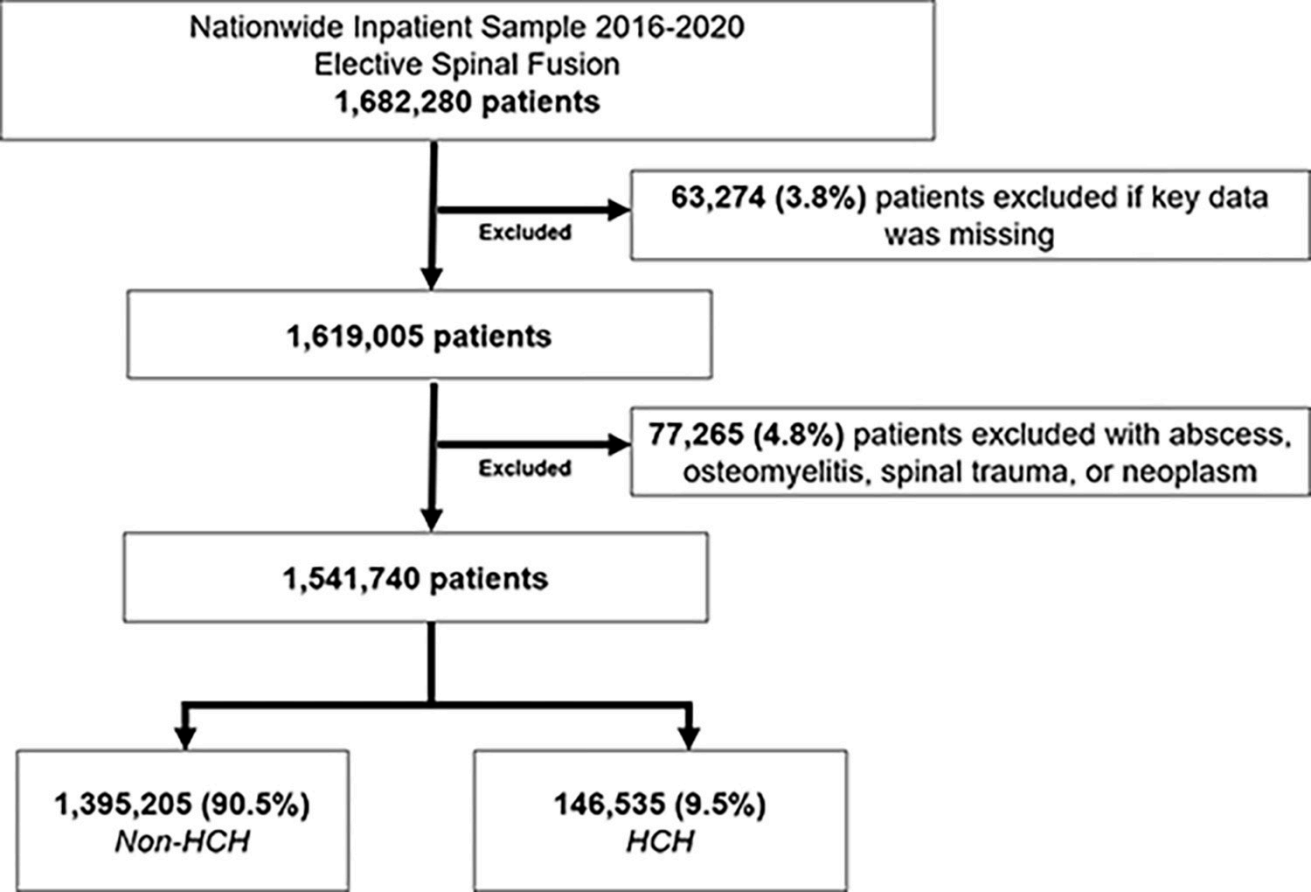
Flow diagram of study cohort of patients undergoing elective spinal fusion at non-high-cost hospitals (Non-HCH) and high-cost hospitals (HCH).

The 2016–2020 National Inpatient Sample (NIS) was queried to identify all elective adult (≥18 years) hospitalizations for cervical, thoracic, lumbar, and sacroiliac spinal fusion operations using relevant International Classification of Disease (ICD), Tenth Revision codes (S1 Table). Maintained by the Healthcare Cost and Utilization Project, the NIS is the largest, all-payer, inpatient database in the U.S. and provides accurate estimates for approximately 97% of all hospitalizations. Patients with diagnoses of spinal abscess, osteomyelitis, neoplasm, or trauma, were not included. Records missing data for age, sex, race, hospitalization charges, or other key variables were excluded (3.8%; Fig 1).

### Variable definitions and study outcomes

Patient and hospital-level characteristics including age, sex, race, income quartile, primary payer, hospital region, teaching status, and bed size, were ascertained according to the NIS data dictionary or from relevant ICD-10 diagnostic and procedural codes [8]. The van Walraven modification of the Elixhauser comorbidity index was used to quantify the burden of chronic conditions [9,10]. Hospitalization costs were calculated by application of institution-specific cost-to-charge ratios to overall charges followed by inflation adjustment to the 2020 Personal Health Index [8,11].

A multi-level, mixed-effects regression model was developed to determine factors associated with cost. Patient characteristics including age, gender, Elixhauser Index and race comprised the first level while individual hospitals were considered as the second level. The interclass correlation coefficient (ICC) was calculated using the “estat icc” command in Stata to determine the proportion of observed variation in cost attributable to center-level differences. Random effects were then estimated by applying validated Bayesian methodology and used to rank hospitals by increasing risk-adjusted costs [12,13]. Centers exceeding the 90^th^ percentile of costs were classified as high-cost hospitals, HCH (Others: non-HCH) [14,15]. This cutoff was determined by visual inspection of the inflection point of calculated risk-adjusted costs in Fig 3.

The primary outcome of interest was the proportion of cost variation attributable to center-level characteristics. Secondary outcomes included major complications, non-home discharge, and length of stay (LOS). Major complications were defined as a composite end point of in-hospital myocardial infarction (MI), blood transfusion, respiratory complications (acute respiratory failure, prolonged ventilation, pneumothorax, and acute respiratory distress syndrome), thrombotic complications (deep vein thrombosis and pulmonary embolism), and infection (sepsis or systemic inflammatory response syndrome). Neurologic (stroke or transient ischemic attack) and cardiac complications (cardiac arrest or myocardial infarction) were additionally analyzed.

### Statistical analysis

Categorical data are reported as group proportions (%) while continuous data are displayed medians with interquartile range (IQR). The adjusted Wald and Pearson χ^2^ tests were used to ascertain the significance of intergroup differences for continuous and categorical variables, respectively. Cuzick’s non-parametric rank-based test was used to examine the significance of temporal trends (*nptrend*) [16]. The Least Absolute Shrinkage Selection Operator (LASSO) was used to guide covariate selection for regression models. This regularization method enhances the accuracy and out-of-sample reliability of prediction models while reducing collinearity among covariates [17]. Multivariable logistic and linear regression models were used to examine the association between covariates and outcomes of interest. All models were optimized using Akaike and Bayesian information criteria and receiver operating characteristics (C-statistic), as appropriate [18]. Regression outputs are reported as adjusted odds ratios (AOR) or beta-coefficients (β) both with 95% confidence intervals (CI).

All statistical analyses were performed using Stata 16.1 software (StataCorp, College Station, TX) with α set at 0.05 for significance. This study was deemed exempt from full review by the Institutional Review Board at the University of California, Los Angeles.

## Results

### Hospital characteristics

From 2016 to 2020, HCH, defined as exceeding the 90^th^ percentile of costs, performed an average of 9.5% of annual spinal fusion cases. Additionally, HCH were more likely to be small (36.8 vs 30.5%, p<0.001), rural (10.1 vs 8.8%, p<0.001), and located in the Western geographic region (49.9 vs 16.7%, p<0.001). Finally, HCH were less frequently classified as urban teaching centers (54.3 vs 58.6%, p<0.001; Table 1).

**Table 1:** Hospital and demographic characteristics of patients undergoing spinal fusion from 2016–2020.

	*Non-HCH*	*HCH*	*P-value*
Hospital Characteristics	(n = 3,888)	(n = 435)	
*Hospital teaching status (%)*			<0.001
Non-Metropolitan	8.8	10.1	
Metropolitan Non-Teaching	32.6	35.6	
Metropolitan Teaching	58.6	54.3	
*Hospital Region (%)*			<0.001
Northeast	14.9	7.8	
Midwest	27.4	12.9	
South	41.0	29.4	
West	16.7	49.9	
*Bed Size (%)*			<0.001
Large	38.5	36.8	
Medium	31.0	26.4	
Small	30.5	36.8	
**Patient Characteristics**	(n = 1,395,202)	(n = 146,535)	
Age (years [IQR])	62 [53–70]	62 [52–70]	p = 0.06
Female (%)	53.8	53.3	0.11
Elixhauser Comorbidity Index (median [IQR])	2 [1–3]	2 [1–3]	0.21
*Race (%)*			<0.001
White	81.7	77.4	
Black	9.0	6.8	
Hispanic	5.6	9.0	
Asian/Pacific Islander	1.1	3.0	
Other	2.6	3.7	
*Insurance coverage (%)*			0.02
Private	38.2	40.3	
Medicare	46.1	44.1	
Medicaid	7.6	7.0	
Other Payer	7.4	7.9	
Uninsured	0.7	0.8	
*Region Fused (%)*			<0.001
Occipital-cervical	0.1	0.1	
Cervical	27.7	25.2	
Cervicothoracic	2.5	2.7	
Thoracic	2.2	3.3	
Thoracolumbar	0.6	0.8	
Lumbar	28.4	36.1	
Lumbosacral	27.7	31.8	
Right sacroiliac joint	2.0	2.5	
Left sacroiliac joint	0.5	0.8	
*Levels Fused (%)*			0.001
1 Level	34.4	26.7	
> 1 Level	65.6	73.3	
*Diagnosis (%)*			<0.001
Herniated Disc	2.5	1.4	
Spinal Stenosis	48.4	45.4	
Spondylolisthesis	39.5	39.5	
Scoliosis	10.0	13.7	

HCH, high-cost hospital; SD, standard deviation; IQR, interquartile range.

### Patient characteristics

**Fig 2:**
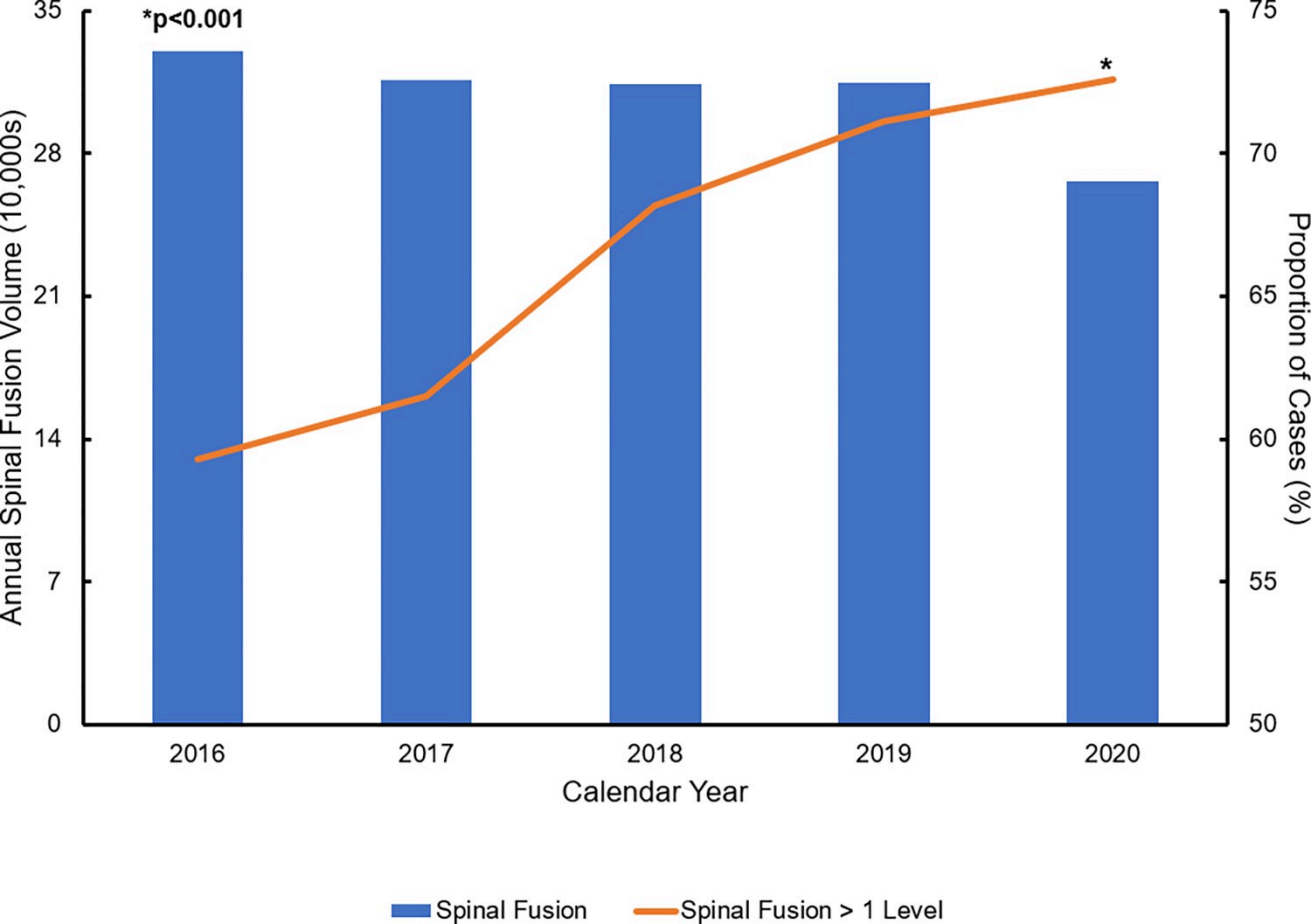
National trends in overall volume of spinal fusion and the proportion of fusions that involved >1 vertebral level, 2016–2020. *nptrend* <0.001.

During the study period, an estimated 1,541,740 patients underwent elective spinal fusion (single-level: 519,685). The overall incidence of spinal fusion decreased while the proportion of operations involving more than one level increased as shown in Fig 2 (nptrend<0.001). There was no significant difference in sex (females 53.3 vs 53.9%, p = 0.11), age (62 [52–70] vs 62 [53–70] years, p = 0.06), or burden of comorbid disease as measured by the Elixhauser Index (2 [1–3] vs 2 [1–3], p = 0.21) between HCH and non-HCH patients. Furthermore, HCH cohort more commonly underwent fusion at more than one spinal level (39.9 vs 36.9%, p<0.001) and for scoliosis (73.3 vs 65.6%, p<0.001) compared to others. Finally, HCH patients were more commonly in the highest income quartile (37.6 vs 21.9%, p<0.001) and more likely to have private insurance (40.3 vs 38.2%, p<0.001; Table 1).

### Interhospital cost variation

**Fig 3:**
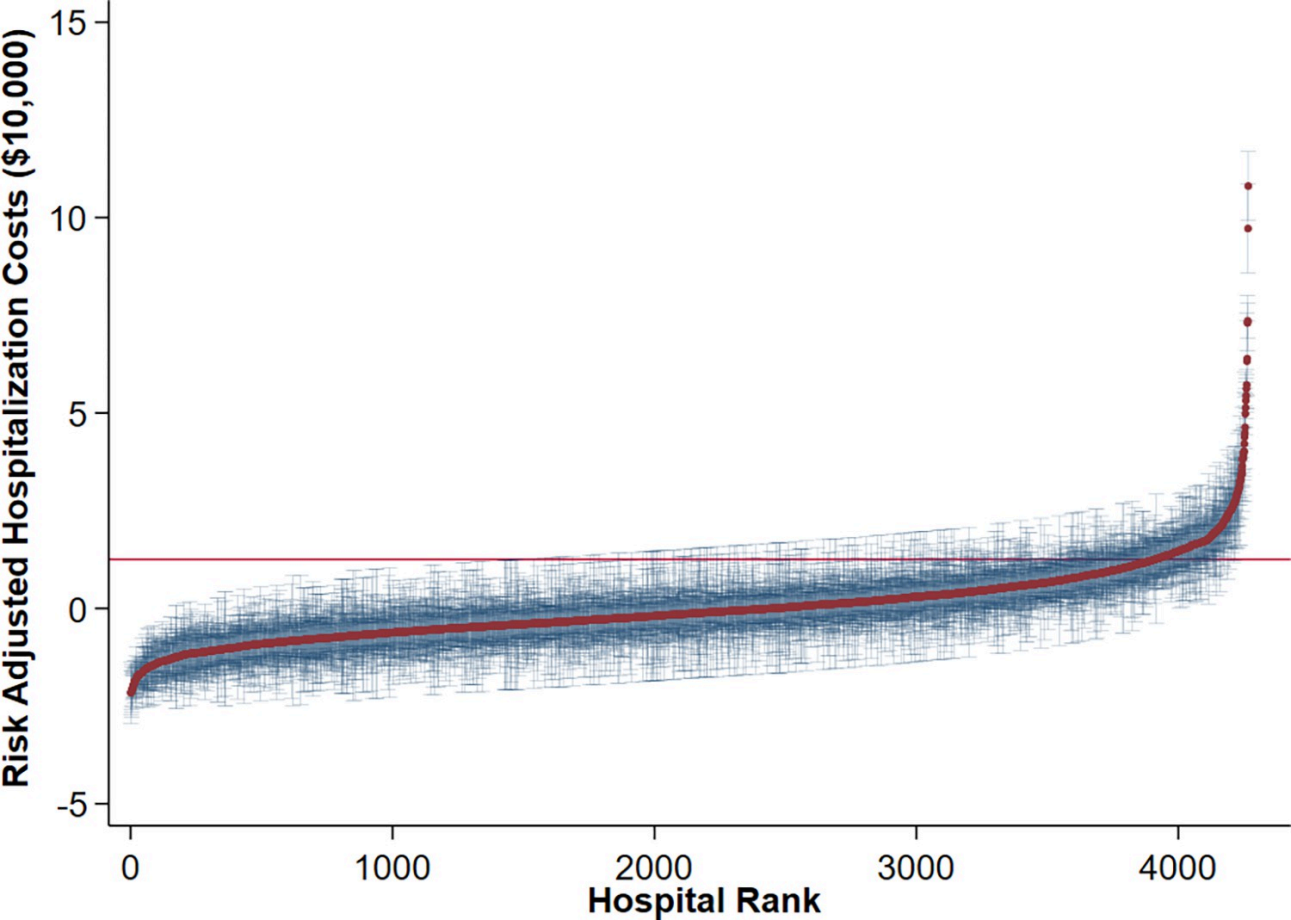
Interhospital variation in risk-adjusted costs.

A multilevel mixed-effects regression was developed to model costs of care. Analysis of the intraclass correlation for the model demonstrated 32% of the explained variation to be attributable to differences among hospitals rather than patient factors (Fig 3).

### Outcomes at high-cost hospitals

On unadjusted analysis, patients treated at HCH had higher hospitalization costs ($47,000 [+32,500, +68,800] vs. $25,200 [+17,600, +36,100], p<0.001) and increased LOS (3 [2–4] vs. 2 [1–3] days, p<0.001; Table 2). Furthermore, HCH had higher hospitalization costs per day ($16,700 [11,500–24,400] vs $10,600 [7,200–15,700], p<0.001) compared to non-HCH.

**Table 2:** Unadjusted outcomes of patients undergoing spinal fusion. HCH, high-cost hospital; *SD*, *standard deviation; IQR*, *interquartile range*.

	*Non-HCH* (n = 1,395,202)	*HCH* (n = 146,535)	*P-value*
**Resource utilization (median [IQR])**			
Cost (USD $10,000)	$25.2 [$17.6 - $36.1]	$47.0 [$32.5 - $68.8]	<0.001
Length of stay (days)	2 [1–4]	3 [2–4]	<0.001
**Complications (%)**			
Mortality	0.09	0.08	0.49
Major Adverse Events	2.1	2.8	<0.001
Neurological	0.33	0.40	0.06
Cardiac	0.46	0.57	0.02
Respiratory	1.67	2.10	<0.001
Thrombotic	0.42	0.63	<0.001
Non-home Discharge	32.7	37.5	<0.001

After adjustment, HCH remained associated with increased hospitalization costs (β $22,900, 95%CI [+21,400, +24,400], p<0.001) and LOS (β 0.34, 95%CI [0.26, 0.42] days, p<0.001). Receiving a spinal fusion at HCH was associated with increased odds of non-home discharge (AOR 1.30, 95%CI [1.17–1.45], p<0.001) and of receiving fusion at >1 level (AOR 1.39, 95%CI [1.30–1.49], p = 0.03), but no increase in the risk of mortality or perioperative complications (Table 3).

**Table 3:** Adjusted results of patients undergoing spinal fusion (Reference Group (REF): *Non-high-cost hospitals); AOR*, *adjusted odds ratio; β*, *beta coefficient; CI*, *confidence interval*.

	*Estimate*	*95% CI*	*P-value*
**Resource utilization (β; REF: Non-HCH)**			
Cumulative Cost (USD $1,000) [IQR]	22.9	[21.4–24.4]	<0.001
Overall length of stay (days) [IQR]	0.34	[0.26–0.42]	<0.001
Multi-level Fusion	1.39	[1.30–1.49]	<0.001
**Complications (AOR; REF: Non-HCH)**			
Mortality	0.74	[0.48–1.15]	0.18
Major Adverse Events	1.04	[0.93–1.16]	0.52
Neurological	1.02	[0.81–1.27]	0.89
Cardiac	1.02	[0.85–1.22]	0.85
Respiratory	0.98	[0.86–1.11]	0.72
Thrombotic	1.09	[0.88–1.36]	0.41
Non-home Discharge	1.30	[1.17–1.45]	<0.001

### Predictors of high-cost

**Fig 4:**
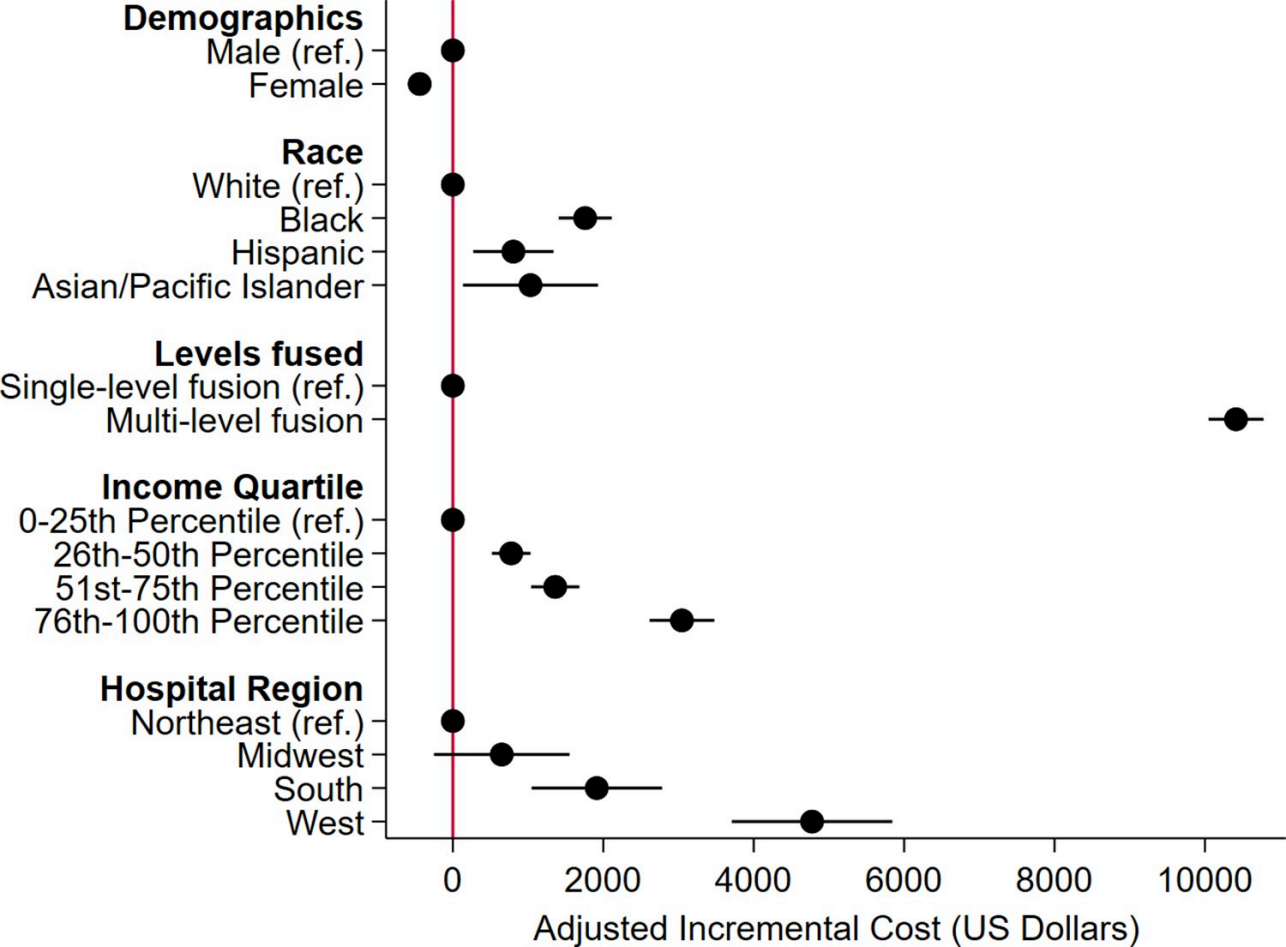
Coefficient plot for incremental costs after spinal fusion. Ref, Reference group.

With White as reference, Black (β +$1,800, 95%CI [$1,400–2,100]) and Hispanic (β +$800, 95%CI $300–1,300]) race were associated with increased costs. Additionally, multilevel fusion was associated with increased costs (β $10,400, 95%CI $10,000–10,800]) compared to single-level fusion. Notably, increased annual center-level operative volume was not associated with a significant difference in cost (Fig 4).

### Discussion

In this retrospective study, we examined temporal trends and costs associated with elective spinal fusion across the United States. Our findings revealed a significant increase in the proportion of multi-level spinal fusions and those performed for scoliosis over the study period. After adjusting for pertinent patient-level factors, we noted nearly 32% of the observed cost variation in spinal fusion to be attributable to center rather than patient factors. Moreover, patients receiving care at high cost hospitals were more likely to undergo multi-level fusion, experience a prolonged length of stay, and be discharged to a non-home setting. Interestingly, no significant associations were found between HCH status and the odds of mortality or perioperative complications. These findings have several important implications towards new patient-centered reimbursement models and warrant further discussion.

The growing emphasis on value-based care systems has changed the landscape of spinal fusion procedures over the past decade. In 2011, the Medicare Coverage Advisory Committee introduced a policy to discourage the use of spinal fusion in indications lacking sufficient evidence for effectiveness [19]. Consequently, the proportion of fusions performed for disc herniations declined significantly, while complex fusions for scoliosis have become more prevalent in recent years [20,21]. Our findings align with these evolving trends, showing a decline in the incidence of single-level fusion and a marked rise in the proportion of multi-level fusions. We also demonstrate a notable variation in case mix with multi-level fusions and operations for scoliosis to be more prevalent at centers designated at HCH. These observed discrepancies underscore the potential disparities within surgeon- and institutional-driven practices on spinal fusion procedures [22]. In order to augment patient care within a value-based care framework, a comprehensive analysis of institutional variations in spinal fusion operations is warranted. Such analysis could facilitate the creation and implementation of nationally standardized spinal fusion care practices aimed at reducing resource utilization while maintaining superior clinical outcomes. Similar strategies have demonstrated success in in colorectal, gynecological, and bariatric surgery through Enhanced Recovery After Surgery (ERAS) protocols [23–25]. The identification of large variation in hospital-level practices and costs is the first step in building evidence-based ERAS policies to maximize care value.

While prior work has reported on the costs of spinal fusion, the influence of hospital factors on cost variation has not been examined thus far. In a national study, Martin and colleagues observed a 152% increase in aggregate hospitalization costs for spinal fusion from 2004 to 2015 [26]. However, the increase in cost has not been uniform, with substantial regional variations in coverage and reimbursement policies influencing care practices. In a study conducted by Zygourakis et. al examining spinal surgery costs between 2001–2013, the Western geographical region was found to be associated with significant higher costs compared to the rest of the US across all studied procedures [27]. As the observed cost relationship was ubiquitous, it is likely there are regional policies or systems-based practices which contribute to greater overall hospitalization costs. One such regional policy is the minimum nurse-to-patient ratio required in acute care hospitals in California, which has been demonstrated to increase costs [27]. In congruence with such regional discrepancies, we further identified HCH to be predominantly located in the West. Perhaps the most important finding of this study is the presence of significant variation in costs of care attributable to hospital characteristics. While center-level factors have been recognized to influence expenditures, varying surgeon enthusiasm and local culture have been proposed as potential drivers of cost in spinal fusion procedures [26]. In an institutional study, Epstein et al. emphasized the impact of surgeon preference on cost disparities, noting a 10-fold variation in instrument charges and a 4.8-fold variation in total charges [28]. While some of the variation in costs may be related to patient complexity and clinical decision-making, it is imperative to mitigate cost discrepancies arising from the surgeon and institutional factors. Identifying variations in cost categories, such as instrumentation and operating room costs, may facilitate the standardization of spinal fusion practices and promote the implementation of value-based spine care.

Post operative length of stay and non-home discharge have previously been analyzed as surrogates for care value in the context of spinal fusion [29,30]. Significant disparities exist in LOS and non-home discharge rates following spinal fusion. Indeed, Khan et al. observed prolonged LOS and higher odds of non-home discharge in black patients undergoing spinal surgery compared to white patients [31]. This is consistent with the findings of the present study as race was independently associated with increased costs. While facets such as race, advanced age, extensive comorbidities and multi-level fusion have been associated with higher rates of non-home discharge and prolonged hospitalization, the contribution of hospital-level factors remains relatively understudied [32,33]. Our present analysis found a significantly higher likelihood of non-home discharge and increased LOS among patients undergoing spinal fusion at HCH. Indeed, patients receiving care at HCH may have greater disease severity and medical complexity as evidenced by the increased proportion of multi-level fusions. However, our findings of high costs at some institutions persisted even after controlling for available relevant characteristics including the number of spinal levels fused. Notably, we found that HCH status did not significantly impact the risk of mortality or perioperative complications. Given comparable clinical outcomes across institutions providing spinal fusion, it is plausible to infer that discharge disposition may also be influenced by local protocols and practices. It is probable that HCH lack standardized care protocols which facilitate expedited discharge following surgery or lack the resources needed to arrange discharge for more medically complex patients. Such disparity emphasizes the need for a collaborative effort between health policy leaders and surgeons to identify areas of improvement, develop evidence-based guidelines, and enhance the overall value of care.

Our study has several important limitations including those inherent to its retrospective nature. Given the administrative nature of the NIS, we are unable to ascertain granular information, including exact vertebral fusion levels, imaging results, and laboratory values. Additionally, the retrospective nature of our study did not allow for establishing causal relationships between HCH status and our clinical outcomes of interest. Furthermore, we are unable to ascertain out-of-pocket costs for each admission and are therefore unable to comment on the degree of financial burden patients experience. Moreover, the NIS does not track specific hospitals over time, limiting our ability to comment on changes in charge practices and spinal fusion utilization within individual hospitals. Despite such limitations, we utilized the largest all payer database in the US and applied robust statistical methods to reduce the risk of bias and enhance generalizability.

## Conclusion

In the present contemporary analysis, the largest all-payer nationally representative database was used to evaluate hospital variation in costs of spinal fusion. Our findings demonstrate hospital-level variation in procedural costs associated with spinal fusion. Additionally, we observed a notable decline in the incidence of spinal fusions, accompanied by a significant increase in the proportion of multi-level fusions. Importantly, our analysis revealed that patients managed at HCH had higher odds of undergoing multi-level fusion, experiencing prolonged LOS and being discharged to a non-home setting. These findings underscore the presence of significant institutional disparities in costs for spinal fusion. Given the implications for care value, a comprehensive investigation of hospital-level variation is essential to optimize resource utilization and improve patient outcomes. Understanding hospital-level variation in costs has important implications for standardizing care in middle- and low-income areas where out-of-pocket medical expenditure is common and contributes to further disadvantage. Further work is needed to elucidate specific institutional practices and barriers that could prevent the successful implementation of standardized care protocols in spinal fusion.

## Supporting information

S1 Table *International Classification of Disease*, *Tenth Revision* (ICD-10) codes for identifying study population. (DOCX)
