# Supplementary material for: Hospital-level variation in hospitalization costs for spinal fusion in the United States
Source: PLoS One. 2024 Feb 8;19(2):e0298135. doi: 10.1371/journal.pone.0298135 (PMC10852221; doi:10.1371/journal.pone.0298135)
Supplement: S1 Table — (DOCX) [file pone.0298135.s001.docx]

**Supplemental Table S1.** *International Classification of Disease, Tenth Revision* (ICD-10) Codes for Identifying Study Population.

| **Spinal Fusion** | **ICD-10 Codes** |
| --- | --- |
| Occipital-cervical | 0RG0070, 0RG0071, 0RG007J, 0RG00J0, 0RG00J1, 0RG00JJ, 0RG00K0, 0RG00K1, 0RG00KJ, 0RG0370, 0RG0371, 0RG037J, 0RG03J0, 0RG03J1, 0RG03JJ, 0RG03K0, 0RG03K1, 0RG03KJ, 0RG0470, 0RG0471, 0RG047J, 0RG04J0, 0RG04J1, 0RG04JJ, 0RG04K0, 0RG04K1, 0RG04KJ |
| Cervical | 0RG1070, 0RG10J0, 0RG10K0, 0RG1370, 0RG13J0, 0RG13K0, 0RG1470, 0RG14J0, 0RG14K0, 0RG1071, 0RG10J1, 0RG10K1, 0RG1371, 0RG13J1, 0RG13K1, 0RG1471, 0RG14J1, 0RG14K1, 0RG2070, 0RG2071, 0RG207J, 0RG20A0, 0RG20AJ, 0RG20J0, 0RG20J1, 0RG20JJ, 0RG20K0, 0RG20K1, 0RG20KJ, 0RG2370, 0RG2371, 0RG237J, 0RG23A0, 0RG23AJ, 0RG23J0, 0RG23J1, 0RG23JJ, 0RG23K0, 0RG23K1, 0RG23KJ, 0RG2470, 0RG2471, 0RG247J, 0RG24A0, 0RG24AJ, 0RG24J0, 0RG24J1, 0RG24JJ, 0RG24K0, 0RG24K1, 0RG24KJ |
| Cervicothoracic | 0RG4070, 0RG40J0, 0RG40K0, 0RG4370, 0RG43J0, 0RG43K0, 0RG4470, 0RG44J0, 0RG44K0, 0RG4071, 0RG40J1, 0RG40K1, 0RG4371, 0RG43J1, 0RG43K1, 0RG4471, 0RG44J1, 0RG44K1 |
| Thoracic | 0RG6070, 0RG60J0, 0RG60K0, 0RG6370, 0RG63J0, 0RG63K0, 0RG6470, 0RG64J0, 0RG64K0, 0RG6071, 0RG60J1, 0RG60K1, 0RG6371, 0RG63J1, 0RG63K1, 0RG6471, 0RG64J1, 0RG64K1, 0RG7070, 0RG7071, 0RG707J, 0RG70A0, 0RG70AJ, 0RG70J0, 0RG70J1, 0RG70JJ, 0RG70K0, 0RG70K1, 0RG70KJ, 0RG7370, 0RG7371, 0RG737J, 0RG73A0, 0RG73AJ, 0RG73J0, 0RG73J1, 0RG73JJ, 0RG73K0, 0RG73K1, 0RG73KJ, 0RG7470, 0RG7471, 0RG747J, 0RG74A0, 0RG74AJ, 0RG74J0, 0RG74J1, 0RG74JJ, 0RG74K0, 0RG74K1, 0RG74KJ |
| Thoracolumbar | 0RGA071, 0RGA0J1, 0RGA0K1, 0RGA371, 0RGA3J1, 0RGA3K1, 0RGA471, 0RGA4J1, 0RGA4K1, 0RGA070, 0RGA0J0, 0RGA0K0, 0RGA370, 0RGA3J0, 0RGA3K0, 0RGA470, 0RGA4J0, 0RGA4K0 |
| Lumbar | 0SG0070, 0SG00J0, 0SG00K0, 0SG0370, 0SG03J0, 0SG03K0, 0SG0470, 0SG04J0, 0SG04K0, 0SG0071, 0SG00J1, 0SG00K1, 0SG0371, 0SG03J1, 0SG03K1, 0SG0471, 0SG04J1, 0SG04K1, 0SG007J, 0SG00JJ, 0SG00KJ , 0SG037J, 0SG03JJ , 0SG03KJ, 0SG047J, 0SG04JJ, 0SG04KJ, 0SG1070, 0SG1071, 0SG107J, 0SG10A0, 0SG10AJ, 0SG10J0, 0SG10J1, 0SG10JJ, 0SG10K0, 0SG10K1, 0SG10KJ, 0SG1370, 0SG1371, 0SG137J, 0SG13A0, 0SG13AJ, 0SG13J0, 0SG13J1, 0SG13JJ, 0SG13K0, 0SG13K1, 0SG13KJ, 0SG1470, 0SG1471, 0SG147J, 0SG14A0, 0SG14AJ, 0SG14J0, 0SG14J1, 0SG14JJ, 0SG14K0, 0SG14K1, 0SG14KJ |
| Lumbosacral | 0SG3070, 0SG30J0, 0SG30K0, 0SG3370, 0SG33J0, 0SG33K0, 0SG3470, 0SG34J0, 0SG34K0, 0SG3071, 0SG30J1, 0SG30K1, 0SG3371, 0SG33J1 , 0SG33K1, 0SG3471, 0SG34J1, 0SG34K1, 0SG307J, 0SG30JJ, 0SG30KJ, 0SG337J, 0SG33JJ, 0SG33KJ, 0SG347J, 0SG34JJ, 0SG34KJ |
| Sacroiliac | 0SG704Z, 0SG707Z, 0SG70JZ, 0SG70KZ, 0SG734Z, 0SG737Z, 0SG73JZ, 0SG73KZ, 0SG744Z, 0SG747Z, 0SG74JZ, 0SG74KZ, 0SG804Z, 0SG807Z, 0SG80JZ, 0SG80KZ, 0SG834Z, 0SG837Z, 0SG83JZ, 0SG83KZ, 0SG844Z, 0SG847Z, 0SG84JZ, 0SG84KZ |
